# Neonatal Acute Kidney Injury

**DOI:** 10.3389/fped.2022.842544

**Published:** 2022-04-07

**Authors:** Cassandra Coleman, Anita Tambay Perez, David T. Selewski, Heidi J. Steflik

**Affiliations:** ^1^Division of Neonatal-Perinatal Medicine, Department of Pediatrics, Medical University of South Carolina, Charleston, SC, United States; ^2^Division of Pediatric Nephrology, Department of Pediatrics, Medical University of South Carolina, Charleston, SC, United States

**Keywords:** acute kidney injury, neonatal, continuous renal replacement therapy, fluid overload, premature (babies), NICU, renal failure, kidney support therapy

## Abstract

Acute kidney injury (AKI) is a common occurrence in the neonatal intensive care unit (NICU). In recent years, our knowledge of the incidence and impact of neonatal AKI on outcomes has expanded exponentially. Neonatal AKI has been shown to be associated with adverse outcomes including increased length of mechanical ventilation, prolonged length of stay, and rise in mortality. There has also been increasing work suggesting that neonates with AKI are at higher risk of chronic kidney disease (CKD). In the past, AKI had been defined multiple ways. The utilization of the neonatal modified Kidney Disease: Improving Global Outcomes (KDIGO) criteria as the standard definition for neonatal AKI in research and clinical care has driven the advances in our understanding of neonatal AKI over the last 10 years. This definition has allowed researchers and clinicians to better understand the incidence, risk factors, and outcomes associated with neonatal AKI across populations through a multitude of single-center studies and the seminal, multicenter Assessment of Worldwide Acute Kidney Injury Epidemiology in Neonates (AWAKEN) study. As the impacts of neonatal AKI have become clear, a shift in efforts toward identifying those at highest risk, protocolizing AKI surveillance, improving prevention and diagnosis, and expanding kidney support therapy (KST) for neonates has occurred. These efforts also include improving risk stratification (identifying high risk populations, including those with nephrotoxic medication exposure) and diagnostics (novel biomarkers and diagnostic tools). Recent work has also shown that the targeted use of methylxanthines may prevent AKI in a variety of high-risk populations. One of the most exciting developments in neonatal AKI is the advancement in technology to provide KST to neonates with severe AKI. In this comprehensive review we will provide an overview of recent work and advances in the field of neonatal AKI. This will include a detailed review of (1) the definition of neonatal AKI, (2) the epidemiology, risk factors, and outcomes associated with neonatal AKI, (3) improvements in risk stratification and diagnostics, (4) mitigation and treatment, (5) advancements in the provision of KST to neonates, and (6) the incidence and risk of subsequent CKD.

## Introduction

Acute kidney injury (AKI) occurs commonly in the neonatal intensive care unit (NICU) and is associated with increase morbidity and mortality. Furthermore, those who develop neonatal AKI may be at increased risk for the development of chronic kidney disease (CKD). With ongoing study, the definition of neonatal AKI has evolved and been standardized, improving our ability to quantify and describe the epidemiology and outcomes associated with neonatal AKI. Here we will review the definition of neonatal AKI, the epidemiology, risk factors, and outcomes associated with neonatal AKI, strategies to improve risk stratification, diagnostics, mitigation, and treatment, recent advances in kidney support therapy (KST) for neonates, and the incidence and risk for CKD following AKI in the NICU.

## Definition

Over the last 20 years the field of critical care nephrology and the study of AKI have been driven by the utilization of agreed upon standardized definitions of AKI. In 2012, Jetton et al. first put forth a modification of the Acute Kidney Injury Network AKI definition and subsequently the Kidney Disease: Improving Global Outcomes (KDIGO) definition was developed and modified for neonates (1). This definition has since been adopted as the neonatal modified KDIGO definition and has served as the consensus definition that should be utilized in research and clinically to diagnose and stage AKI (Table 1) (2, 3). This definition was agreed upon as the consensus definition of neonatal AKI at a multidisciplinary 2013 National Institutes of Health workshop dedicated to neonatal AKI (2). The neonatal, modified KDIGO definition represents the first consensus definition of neonatal AKI and has been validated in the international multicenter, Assessment of Worldwide Acute Kidney Injury Epidemiology in Neonates (AWAKEN) study (1).

**TABLE 1:** Neonatal acute kidney injury diagnostic criteria.

AKI stage	Serum creatinine (SCr) criteria	Urine output criteria (hourly rate)
0	No change in SCr *or* SCr rise < 0.3 mg/dL	≥0.5 ml/kg/h
1	SCr rise ≥ 0.3 mg/dL rise within 48 h *or* SCr rise ≥ 1.5–1.9 × baseline SCr^a^	<0.5 ml/kg/h × 6–12 h
2	SCr rise ≥ 2.0–2.9 × baseline SCr^a^	<0.5 ml/kg/h for >12 h
3	SCr rise ≥ 3 × baseline SCr^a^ *or* SCr ≥ 2.5 mg/dL^b^ *or* Kidney support therapy utilization	<0.3 ml/kg/h for ≥24 h *or* Anuria for ≥12 h

*Modified, neonatal Kidney Disease: Improving Global Outcomes (KDIGO) criteria. ^a^Baseline SCr defined as lowest previous SCr value. ^b^SCr value of 2.5 mg/dL represents glomerular filtration rate of <10 mL/min/1.73 m^2^. SCr, serum creatinine; mg/dL, milligrams per deciliter; h, hours. Adapted from Kidney Disease: Improving Global Outcomes (KDIGO) Acute Kidney Injury Workgroup. KDIGO clinical practice guideline for acute kidney injury. Kidney Int Suppl 2012;2:1–138.*

While the development and utilization of the neonatal modified KDIGO definition has driven the advancements in the field over the last 5–10 years, it has also become clear that there are short-comings of the current definition that will warrant future refinement. The most obvious issues relate to utilizing the current thresholds of changes in serum creatinine (SCr) to define neonatal AKI. A recent secondary analysis of the AWAKEN study shows that the ideal definition of neonatal AKI may not be the same across gestational age (GA) groups (4). Similarly, work by Gupta et al. interrogated the impact of the failure of SCr to drop over the first postnatal week, which does not qualify as AKI by the current neonatal modified KDIGO definition (5). In this study of 106 neonates with hypoxic ischemic encephalopathy (HIE), a failure of SCr to drop by 50% or fall below 0.6 mg/dL was associated with adverse outcomes. Additionally, the optimal definition of oliguria that defines AKI in neonates and the incorporation of novel biomarkers (discussed later) into the definitions of AKI remain active areas of research need in neonatal AKI. As with all definitions in medicine, the definition of neonatal AKI represents an iterative process that will be refined over time with future consensus conferences. At this time, the neonatal modified KDIGO definition remains the gold-standard to define neonatal AKI for clinical and research purposes.

## Epidemiology, Risk Factors, and Outcomes of Neonatal AKI

To understand the unique epidemiology, risk factors, and outcomes associated with neonatal AKI, it is important to appreciate the nuances of neonatal renal development and physiology. Neonatal kidneys are particularly susceptible to hypoperfusion and ischemia secondary to the dynamic changes in renal blood flow that occur postnatally. These perfusion alterations are driven by changes in the renin-angiotensin system and prostaglandins making neonates susceptible to medications such as angiotensin-converting enzyme (ACE) inhibitors and non-steroidal anti-inflammatory drugs (NSAIDS).

*In utero* nephron development begins at 5 weeks’ gestation and continues to 34–36 weeks of gestation (6). The majority of nephrogenesis occurs late in pregnancy with up to 60% of nephrogenesis occurring in the third trimester (6, 7). While final nephron number is highly variable, each additional kilogram (kg) increase in birth weight (BW) confers nearly 200,000 additional nephrons (8–10). As a result, premature birth and low birth weight (LBW) both alter final nephron number and development increasing the risk for AKI and subsequent CKD (11–18). The neonatal kidney continues to mature over the first two postnatal years as renal vascular resistance falls, cardiac output to the kidney increases, and the adult-level glomerular filtration rate (GFR) is established (19).

Over the last decade, our understanding of the epidemiology of neonatal AKI has expanded exponentially with the utilization of the neonatal modified KDIGO consensus definition of neonatal AKI. In the largest study to date, the AWAKEN study, investigators evaluated the incidence and impact of neonatal AKI in an international, multicenter cohort study of 2022 neonates admitted to 24 NICUs over a 3-month period. In this study, incidence of AKI was 30%, with variation by GA (≥22 to <29 weeks’: 48%; ≥29 to <36 weeks’: 18%; ≥36 weeks’: 37%) (1). This study showed that AKI in neonates was independently associated with increased mortality (9.7 vs. 1.4%; *p* < 0.001; adjusted odds ratio (aOR) 4.6 [95% confidence interval (CI) 2.5–8.3; *p* < 0.0001] and length of stay [adjusted parameter estimate 8.8 days (95% CI 6.1–11.5); *p* < 0.0001] after adjustment for 16 variables.

Though the incidence of neonatal AKI is high among NICU patients, specific sub-populations are at particularly high risk and warrant further discussion (Table 2). Several of these populations are discussed in detail below.

**TABLE 2:** Epidemiology of high risk populations for neonatal acute kidney injury.

NICU sub-population	Study details	AKI incidence	Significant findings
Premature and low birth weight (LBW) neonates	Hingorani et al. (*n* = 900) (14)	19% severe AKI^†^	• Stage 3: AKI occurring 7 days before death independently associated with death • Severe AKI associated with increased hazard of death
	Askenazi et al. (*n* = 923) (13)	38%	• 18% had 1 episode of severe^†^ AKI • Rates of severe^†^ AKI: ° 24 weeks GA: 27.8% ° 25 weeks GA: 21.9% ° 26 weeks GA: 13.6% ° 27 weeks GA: 9.4% • AKI rates significantly higher with decreasing GA and BW
	Lee et al. (*n* = 276) (15)	56% (Stage 1: 30%; Stage 2: 17%; Stage 3: 9%)	• High-frequency ventilation support, PDA, lower GA, and inotropic agent utilization independently associated with AKI • Maternal pre-eclampsia is protective against neonatal AKI • AKI associated with higher mortality before 36 weeks PMA
	Carmody et al. (*n* = 455) (20)	39.8% (16.5% multiple episodes)	• GA < 28 weeks’ associated with AKI • AKI independently associated with increased mortality and increased length of hospital stay
	Koralkar et al. (*n* = 229) (87)	18%	• AKI was independently associated with increased mortality
	Askenazi et al. (*n* = 195) (88)	Case–control study	
Congenital heart disease (CHD) and cardiac surgery	Sasaki et al. (*n* = 582) (89)	38%	• AKI prevalence peaked on post-operative day 1 (17%) • No stage of AKI was associated with ventilation hours or length of stay
	Alten et al. (*n* = 22,400) (26)	CS-AKI 53.8% (Stage 1: 31%; Stage 2: 13.5%; Stage 3: 9.1%)	• CS-AKI varied greatly across institutions • Pre-operative enteral feeding and open sternum were associated with less CS-AKI • CPB was associated with increased CS-AKI • Stage 3: CS-AKI independently associated with mortality
	Alabbas et al. (*n* = 122) (90)	62%	• Severe AKI (stage 3) was independently associated with increased mortality and length of stay
	Blinder et al. (*n* = 430) (22)	52% (Stage 1: 31%; Stage 2: 14%; Stage 3: 7%)	• Single ventricle status, CPB, and higher reference SCr were associated with post-operative AKI • Post-operative AKI (all stages) associated with longer intensive care stay • More severe AKI associated with in-hospital mortality, longer duration of mechanical ventilation, longer duration of inotropic support • Stage 3: AKI associated with systemic ventricular dysfunction at hospital discharge
Hypoxic ischemic encephalopathy (HIE)	Kirkley et al. (*n* = 113) (91)	41.6%	• Outside hospital birth, IUGR, and meconium at delivery associated with increased odds of AKI • Infants with AKI had longer duration of stay compared to those without AKI
	Chock et al. (*n* = 38) (92)	39%	• Those with AKI had higher renal artery saturations (Rsat; *via* NIRS) compared to those without AKI after 24 h of life • Rsat > 75% by 24–48 h predicted AKI with sensitivity 79% and specificity 82% (AUC 0.76)
	Tanigasalam et al. (*n* = 120) (35)	32% in TH; 60% in standard tx	AKI incidence in TH vs. standard tx groups: • Stage 1: 22 vs. 52% • Stage 2: 5 vs. 5% • Stage 3: 5 vs. 3%
	Sarkar et al. (*n* = 88) (33)	39%	• AKI independently associated with abnormal brain MRI
	Selewski et al. (*n* = 96) (28)	38%	• AKI predicted prolonged duration of mechanical ventilation and length of stay
Necrotizing enterocolitis (NEC)	Garg et al. (*n* = 202) (36)	32.6% severe AKI NEC dx; 58.7% after surgical NEC	• Surgical NEC, outborn status, exposure to antenatal steroids, and positive blood culture sepsis had increased odds of severe AKI • Severe AKI associated with longer duration of hospitalization
	Bakhoum et al. (*n* = 77) (39)	42.9% (Stage 1: 18.2%; Stage 2: 13%; Stage 3: 11.7%)	• Bell’s stage II NEC with AKI: 63.6% • Bell’s stage III NEC with AKI: 36.4% • Bell’s Stage III NEC, lower GA, maternal pre-eclampsia/eclampsia, gentamicin/vancomycin exposure, and empiric antibiotic use independently associated with AKI • AKI independently associated with mortality
	Criss et al. (*n* = 181) (38)	54% (Stage 1: 22%; Stage 2: 18%; Stage 3: 16%)	• Neonates with AKI had higher mortality and higher chance of death
Nephrotoxic medications (NTX)	Salerno et al. (*n* = 8,286) (43)	17%	• Sepsis, lower baseline SCr, and duration of combination therapy were associated with increased odds of AKI • [Furosemide + tobramycin] and [vancomycin + piperacillin-tazobactam] were associated with decreased risk of AKI relative to [gentamicin + indomethacin]
	Rhone et al. (*n* = 107) (42)	Infants with AKI received more NTX per than those without AKI	• Exposure to 1 NTX occurred in 87% of VLBW infants ° Gentamicin: 86% ° Indomethacin: 43% ° Vancomycin: 25% • Inverse relationship between BW and NTX received per day
Extracorporeal Life support (ECLS)	Murphy et al. (*n* = 446) (49)	51%	• AKI most common in those with cardiac disease but varies by underlying diagnosis • Risk of mortality differed by diagnostic category in the presence or absence of AKI ° Without AKI, CDH independently predicts mortality
	Fleming et al. (*n* = 832) (48)	74%	• AKI during ECLS was associated with longer duration of ECLS support and increased adjusted odds for mortality
	Zwiers et al. (*n* = 242) (45)	64%	• Increased risk of mortality at highest stage of AKI

*NICU, neonatal intensive care unit; AKI, acute kidney injury; GA, gestational age; BW, birth weight; PDA, patent ductus arteriosus; PMA, post menstrual age; CS-AKI, cardiac surgery-associated AKI; CPB, cardiopulmonary bypass; SCr, serum creatinine; IUGR, intrauterine growth restriction; Rsat, renal saturations; NIRS, near-infrared spectroscopy; AUC, area under the curve; TH, therapeutic hypothermia; MRI, magnetic resonance imaging; NEC, necrotizing enterocolitis; NTX, nephrotoxic medication; CDH, congenital diaphragmatic hernia; ECLS, extracorporeal life support; tx, treatment. ^†^Severe AKI defined as = stage 2 AKI.*

### Prematurity and Low Birth Weight

There has been a considerable amount of work highlighting the high incidence and impact of AKI in premature infants. This work has confirmed a significant association between LBW, early GA, and AKI (11–16). In a single retrospective study of 455 very LBW neonates (VLBW, i.e., ≤1,500 g), Carmody et al. showed that AKI occurred in 40% of neonates with 16.5% of infants experiencing multiple episodes of AKI (20). In this cohort, AKI was associated with increased mortality (aOR, 4.0; 95% CI 1.4–11.5) and length of stay (11.7 days; 95% CI 5.1–18.4 days). This group also noted that AKI was only present as a diagnosis in 13.5% of discharges, highlighting the need for continued education of providers. More recently, a secondary analysis of the Preterm Erythropoietin Neuroprotection Trial evaluated the incidence and impact of neonatal AKI in a cohort of 932 extremely low GA neonates (13). In this study the incidence of AKI was 38% and differed significantly by GA, with AKI rates being significantly higher with decreasing GA and BW. In 2021, Wu et al. published a meta-analysis of 50 articles including over 10,000 premature and LBW neonates evaluating the incidence and impact of neonatal AKI (21). This study showed a pooled incidence of AKI of 25% (95% CI 20–30%), and those with AKI had significantly higher odds of mortality (OR 7.1; 95% CI 5.9–8.6; *p* < 0.01).

### Congenital Heart Disease

Neonates and infants undergoing cardiac surgery are among the highest risk patients for developing AKI (22, 23). This results from the complex interaction of a multitude of risk factors including but not limited to low cardiac output, single ventricle physiology, ischemic-reperfusion injury, fluid overload (FO), and nephrotoxic medication (NTX) receipt (17, 24, 25). Despite these factors, the incidence and impact of AKI in this population remains varied in the literature. In a single center retrospective study of 430 infants < 90 days of age undergoing surgical correction, AKI occurred in 52% of the cohort (22). In this study, stage 2 and 3 AKI were associated with increased mortality. More recently the Neonatal and Pediatric Heart and Renal Outcomes in Newborns (NEPHRON) collaborative evaluated the incidence and impact of AKI in a multicenter (22 centers) cohort of neonates (*n* = 2,240) undergoing congenital heart surgery (26). The overall incidence of AKI in this study was 53.8%, and the prevalence of AKI peaked on postoperative day 1; only stage 3 AKI was associated with increased mortality (aOR 2.44; 95% CI 1.3–4.61). A novel finding in this study is the incidence of AKI amongst infants undergoing cardiac surgery varied by institution from 27 to 86%, and a significant amount of AKI was transient in nature. The authors suggest the current definition of neonatal AKI may need to be adjusted to capture truly meaningful phenotypes of cardiac surgery-associated AKI in neonates.

### Hypoxic Ischemic Encephalopathy

Neonates with perinatal asphyxia, also known as HIE, often develop multiorgan failure, which impacts virtually every organ system. AKI has been shown to occur commonly in infants with HIE with an incidence ranging from 38 to 72% (27–32). In a single center study of 96 neonates with HIE undergoing therapeutic hypothermia, AKI occurred in 38% of neonates and independently predicted prolonged duration of mechanical ventilation and NICU stay (28). In a follow-up study, AKI during therapeutic hypothermia was found to be associated with the development of abnormal magnetic resonance imaging (MRI) findings at 7–10 postnatal days (33). The impact of therapeutic hypothermia on the incidence of AKI in HIE remains unclear, with trials and retrospective studies demonstrating conflicting results (34, 35).

### Necrotizing Enterocolitis

Necrotizing enterocolitis (NEC) occurs in 2–5% of NICU admissions and represents a significant cause of morbidity and mortality. Infants with NEC have multiple risk factors for AKI including sepsis, hemodynamic instability, NTX receipt, systemic inflammation, increased intrabdominal pressure, and frequently prematurity (36, 37). In a single center retrospective study of 202 neonates with NEC, the overall incidence of severe AKI was 32.6% and in those requiring surgical intervention, the incidence was 58.7% (36). This study also showed that severe AKI was associated with a longer length of stay {124 days [interquartile range (IQR) 88–187] vs. 82 days (42–126); *p* < 0.001}. Criss et al. reported a similar incidence of any AKI of 54% in a single center cohort of 181 neonates with NEC (38). Studies in neonates with NEC have consistently shown that AKI occurs commonly and is associated with increased morbidity and mortality (11, 38, 39).

### Nephrotoxic Medications

Critically ill neonates are frequently exposed to NTX during their NICU stay. Table 3 outlines the mechanism and nephrotoxicity of commonly encountered NTX in neonates. In older children NTX exposure has been shown to be a potentially modifiable cause of AKI (40, 41). As a result, there has been an increased interest in understanding the epidemiology of NTX exposures in neonates. Rhone et al. evaluated 107 VLBW neonates and described the epidemiology of NTX exposure (42). In this study 87% of all neonates received at least one NTX, with gentamicin (86%), indomethacin (43%) and vancomycin (25%) most commonly administered. Neonates in this study received NTX for a median 8 days (IQR 3–21) with a significant difference in mean days of NTX exposure in those with AKI compared to those without AKI (23.9 vs. 9.9 days; *p* < 0.001).

**TABLE 3:** Nephrotoxic medications frequently used in neonates.

Medication	Mechanism of action	Site of kidney damage	Nephrotoxicity	Notes
Acyclovir	Inhibits DNA synthesis and viral replication *via* inhibition of viral DNA polymerase	Tubule	Crystallization and obstruction occur causing tubular damage, particularly when in low urinary flow state	Can be used for prophylaxis (CMV, HSV, varicella, herpes zoster), suppression (HSV), and treatment (varicella zoster, herpes zoster, HSV, varicella). Dosage adjustment for renal impairment available (93).
Amikacin	Inhibits protein synthesis *via* binding to 30S ribosomal subunits	Proximal tubule, S1 and S2 segments, late changes in S3	Proximal tubular damage after accumulation of aminoglycoside	Dosage adjustment for renal impairment as well as augmented renal clearance available (93).
Amphotericin B	Disrupts fungal cell wall synthesis and cell membrane permeability *via* binding to ergosterol which causes leakage of cellular components and subsequent cell death	Distal tubule	Vasoconstriction and direct distal tubular toxicity	Hydration and sodium repletion prior to administration of amphotericin B may reduce risk of renal toxicity. Dosage adjustment for renal impairment available (93).
Gentamicin	Disrupts bacterial protein synthesis and cell membrane integrity *via* biding to 30S ribosomal subunit	Proximal tubule, S1 and S2 segments, late changes in S3	Proximal tubular damage after accumulation of aminoglycoside	Dosage adjustment for renal impairment available (93).
Indomethacin	Non-selective cyclooxygenase inhibitor decreasing prostaglandin synthesis	Afferent arteriole	Hemodynamically mediated: causes afferent arteriole vasoconstriction and reduced GFR	Dosage adjustment for renal impairment available (93).
Piperacillin/Tazobactam	Inhibits bacterial cell wall synthesis leading to bacteria lysis	Tubule, particularly proximal tubule	Inhibits tubular secretion and clearance, direct toxicity	Dosage adjustment for renal impairment available (93).
Vancomycin	Inhibits cell wall synthesis of gram-positive bacteria *via* blocking glycol-peptide polymerization	Proximal tubule	Direct toxicity, otherwise unclear	Dosage adjustment for renal impairment available (93).

*DNA, deoxyribonucleic acid; CMV, cytomegalovirus; HSV, herpes simplex virus; GFR, glomerular filtration rate.*

Since the publication of this seminal work, there have been further studies evaluating NTX exposure in neonates. In 2021, Salerno et al. evaluated the impact of combinations of NTX in a database including 268 NICUs (43). In this study of 8,286 neonates exposed to NTX, the incidence of AKI was 17%, and increased duration of NTX exposure was associated with increased risk of AKI. An interesting finding in this study is that 23,399 neonates were exposed to NTX during the study period, but 15,113 neonates were excluded from the analysis because they did not have 2 SCr measured. This highlights the potential need for improved surveillance strategies, such as Nephrotoxic Injury Negated by Just-in-time Action (NINJA) (discussed below) (44).

### Extracorporeal Life Support

Neonates treated with extracorporeal life support (ECLS) are the sickest patients in the NICU and are at increased risk of AKI for a multitude of reasons including hypotension, underlying disease etiology, systemic inflammation, hemolysis and hemoglobinuria, micro-emboli, non-pulsatile flow, and NTX exposure. The incidence of AKI in this population is as high as 70% (45–48). In a retrospective single-center study of 242 neonates treated with ECLS, Zwiers et al. found the incidence of AKI was 64% with the most severe stage of AKI associated with increased mortality (45). In a recent report from the multicenter Kidney Interventions During Membrane Oxygenation (KIDMO) study group about 446 neonates treated with ECLS, the incidence of AKI in the overall cohort was 51%, but the AKI incidence varied by underlying diagnosis (cardiac 68%, congenital diaphragmatic hernia 38%, respiratory 33%) (49). The association of AKI with outcomes varied significantly by underlying diagnosis as well.

### Impact of Acute Kidney Injury on Other Organ Systems

Across medicine, the paradigm surrounding AKI has shifted from an isolated disease process impacting one organ to a multisystem disease process that impacts distant organs. Recent work in neonates has begun to highlight this. Studies in neonates report associations between neonatal AKI and intraventricular hemorrhage (IVH) and abnormal brain MRI (33, 50). In a secondary evaluation of 866 premature neonates from the AWAKEN study, AKI was associated with increased odds of IVH (aOR, 95% CI 1.04–2.56) (50). Multiple recent single center studies have confirmed this association of AKI with IVH in premature infants (51, 52). AKI during HIE has also been shown to be associated with increased odds of abnormal brain MRI findings at 7–10 postnatal days (33). In a recent 2-year follow-up study of 101 neonates with HIE, AKI was associated with an unfavorable outcome (death or disability according to Griffiths Mental Development Scales) at 24 months (53).

Recent work has also begun to establish a link between AKI in neonates and respiratory outcomes including length of mechanical ventilation and bronchopulmonary dysplasia (BPD). Starr et al. reported findings from the AWAKEN study which showed that neonates 29–32 weeks’ GA with AKI were more likely to have a poor composite outcome of moderate to severe BPD and/or death (aOR 4.2, 95% CI: 2.1–8.6; *p* < 0.001) (54). This confirmed previous single center work by Askenazi et al. which showed a higher risk of oxygen requirement or of dying at 28 days of life [relative risk (RR) 1.7, 95% CI 1.2–2.4; *p* < 0.002] in a single center cohort of 122 premature infants with AKI (55).

## Advances in Diagnosis, Prevention and Mitigation, and Treatment of Sequelae

There are currently no proven treatments for established AKI. Despite multiple clinical trials across critical care nephrology, no therapeutic interventions have been shown to be effective in patients once AKI has occurred. As a result, efforts to advance the field have shifted toward improved diagnostics, prevention and mitigation strategies, and treatment of sequelae in neonatal AKI.

### Diagnostics

SCr is currently the “gold-standard” of biomarkers to identify AKI, but there are a multitude of challenges with SCr as a biomarker. Most importantly, SCr serves as a measure of kidney function, rather than injury (56). Furthermore, SCr is a delayed (up to 48–72 h) marker of kidney function, which may not change until 25–50% of the kidney function has been lost (6, 57). These impediments taken together may explain the challenges faced with the development of successful clinical trials and interventions in AKI. Efforts to detect AKI earlier have led to the development of novel biomarkers that lead to the timely diagnosis of AKI, improved clinical trials, and improved outcomes.

These novel biomarkers were identified and developed initially in high-risk populations such as those undergoing cardiac surgery, where the incidence of AKI is high and the timing of the insult is known. Many of these biomarkers show promise in the early and accurate diagnosis of neonatal AKI (Table 4). Neutrophil gelatinase associated lipocalin (NGAL) is perhaps the most studied novel biomarker in neonates and as described in Table 4, may predict AKI earlier than changes in SCr in a variety of neonatal populations. Studies to validate its use are ongoing, and consensus on how best to utilize NGAL is lacking. Understanding how to best utilize these novel biomarkers in clinical practice is the subject of ongoing research.

**TABLE 4:** Neonatal acute kidney injury biomarkers.

Biomarker	Properties and production	Notable Studies	
		
		Authors and year	Findings
Cystatin C (CysC)	Cysteine protease produced at a constant rate by all nucleated cells	Hidayati et al. (94)	• Cys-C based estimated GFR to diagnose AKI ° Sensitivity: 84.8% ° Specific: 61.8% ° PPV: 41.8% ° NPV: 89.7% • AUC for CyC: 84.9% • Optimal cut-off for CysC: 1.605 mg/L
		Lagos-Arevalo et al. (95)	• Early ICU CysC predicted SCr-based AKI development • AUC 0.70; 95% CI 0.53–0.89
		Li et al. (96)	• uCysC independently associated with AKI • OR 2.07, AUC 0.92
		Sarafidis et al. (97)	• Asphyxiated neonates had significantly higher ° sCysC on DOL 1 (2.86 mg/L (IQR 2.1–3.0) vs. 2.23 (1.75–2.62); *p* = 0.049) ° uCysC at all time points Compared to non-asphyxiated infants • Urine CysC cut-off > 476 ng/mg: ° AUC 0.927, *p* < 0.001 ° Sensitivity 100% ° Specificity 83.3% • Urine CysC cut-off > 204.4 ng/mL: ° AUC 0.937, *p* < 0.001 ° Sensitivity 100% ° Specificity 91.7%
		Askenazi et al. (98)	Maximum CysC levels did not differ between those with and without AKI nor between survivors and non-survivors
Neutrophil gelatinase-associated lipocalin (NGAL)	Protein expressed by multiple tissues including kidney	Sarafidis et al. (99)	• uNGAL significantly higher in those with AKI compared to those without AKI on day AKI diagnosed by SCr • uNGAL had no significant ability to predict AKI in 1–2 days prior to AKI development
		Tabel et al. (100)	• Median uNGAL significantly higher in preterm infants with AKI than those without AKI on DOL 1 and 7 • uNGAL independently associated with AKI
		Sarafidis et al. (97)	• Asphyxiated neonates had significantly higher sNGAL and uNGAL at all time points compared to non-asphyxiated neonates • sNGAL was significantly higher in asphyxiated infants with AKI compared to non-asphyxiated neonates at all time points, asphyxiated infants with AKI and asphyxiated infants without AKI on DOL 1 and 3 • Serum NGAL cut-off > 89.6 ng/mL: ° AUC: 0.942, *p* < 0.001 ° Sensitivity 100% ° Specificity 92.3% • uNGAL was significantly higher in asphyxiated infants with AKI compared to non-asphyxiated infants at all time points and in asphyxiated infants without AKI and non-asphyxiated infants at DOL 10 • uNGAL cut-off > 39.3 ng/mg: ° AUC 0.896, *p* < 0.001 ° Sensitivity 100% ° Specificity 83.3% • uNGAL cut-off > 18.61 ng/mL: ° AUC 0.865, *p* < 0.001 ° Sensitivity 100% ° Specificity 83.3%
		Krawczeski et al. (101)	• In term neonates requiring CPB, pNGAL and uNGAL significantly higher at 2 h after CPB and remained elevated for 48 h post-operatively in patients with AKI • NGAL 2-hour after CPB the earliest and strongest predictor of AKI
		Askenazi et al. (98)	• Compared to those without AKI, those with AKI had higher max NGAL • AKI: 985 ng/mL (95% CI 452, 1,398) • No AKI: 458 ng/mL (95% CI 210, 587) • For every 100 ng/mL rise in NGAL, the odds of AKI increased by 20% ° OR 1.2 (1.0–1.6), *p* < 0.01 ° AUC 0.80 • Combining NGAL and OPN improved ability to detect AKI ° AUC 0.90 • No difference in NGAL concentrations between survivors and non-survivors
Interleukin-18 (IL-18)	Pro-inflammatory cytokine induced in proximal tubule after AKI and renal tubular injury	Li et al. (96)	• uIL-18 independently associated with AKI in non-septic critically ill neonates ° OR 2.27, AUC 0.72
		Askenazi et al. (98)	Maximum IL-18 levels did not differ between those with and without AKI nor between non-survivors vs. survivors
Kidney injury molecule-1 (KIM-1)	type 1 transmembrane protein that has been found to be highly upregulated in the proximal tubule epithelial cells; secreted in urine after AKI	Askenazi et al. (98)	• Maximum KIM-1 levels did not differ between those with and without AKI • Compared to survivors, non-survivors had higher KIM-1 ° Non-survivors: 385 pg/mL (95% CI 231, 1,028) ° Survivors: 264 (95% CI 147, 549) ° For every 100 pg/mL rise in KIM-1, there was a 10% higher odds of death (OR 1.1 (1.0–1.2), *p* < 0.02; AUC 0.64)
		Sarafidis et al. (97)	• Higher absolute uKIM-1 levels in asphyxiated neonates on DOL 10 • uKIM-1 was comparable between those with asphyxia and AKI, those with asphyxia but no AKI, and non-asphyxiated infants at all time points
Osteopontin (OPN)	Cytokine expressed and upregulated during inflammation and AKI	Askenazi et al. (98)	• Compared to subjects without AKI, those with AKI had higher OPN ° AKI: 468 ng/mL (95% CI 247, 655) ° No AKI: 217 ng/mL (95% CI 115, 280) ° For every 100 ng/mL rise in OPN, the odds of AKI increased by 220% (OR 3.2 (1.5–9.9), *p* < 0.01; AUC 0.83) ° Combining NGAL and OPN improved ability to detect AKI (AUC 0.90) • Compared to survivors, non-survivors had higher maximum OPN ° Non-survivors: 482 ng/mL (95% CI 281, 631) ° Survivors: 20 ng/mL (95% CI 112, 371) ° For every 100 ng/mL rise in OPN, there was a 80% higher odds of death (OR 1.8 (1.2–2.7), *p* < 0.001; AUC 0.78)
Beta-2 microglobulin (B2mG)	Peptide produce from cellular membrane turnover, particularly elevated with tubular dysfunction or injury	Abdullah et al. (102)	• In term asphyxiated neonates, uB2mG levels were significantly higher in infants with AKI compared to those without AKI and were found to be predictive of AKI within the first 24 h after asphyxiation ° AKI: 6.8 mg/L vs. no AKI: 2.6 mg/L, *p* < 0.001 ° AUC: 0.944 ° Ideal cut off: 3.8 mg/L • 81% sensitive • 81.6% specific
		Askenazi et al. (98)	Maximum B2mG levels did not differ between those with and without AKI nor between non-survivors vs. survivors

*GFR, glomerular filtration rate; AKI, acute kidney injury; PPV, positive predictive value; NPV, negative predictive value; AUC, area under the curve; mg/L, milligrams per liter; ICU, intensive care unit; SCr, serum creatinine; uCysC, urine Cystatin C; sCysC, serum Cystatin C; DOL, day of life; IQR, interquartile range; uNGAL, urine neutrophil gelatinase lipocalin; sNGAL, serum neutrophil gelatinase lipocalin; CPB, cardiopulmonary bypass; pNGAL, plasma neutrophil gelatinase lipocalin; h, hours; ng/mL, nanograms per milliliter; CI, confidence interval; uIL-18, urine interleukin-18; OR, odds ratio; pg/mL, picograms per milliliter; uKIM-1, urinary kidney injury molecule-1; uB2mG, urine Beta-2 microglobulin.*

### Prevention and Mitigation

#### Risk Stratification

A critical step in improving outcomes in AKI is the early identification of populations that are at the highest risk for the development of AKI. The concept of risk stratification is an active area of research across critical care nephrology and is embodied by the concept of “renal angina.” In pediatric patients, the “Renal Angina Index” has been developed based on clinical risk factors and signs of injury. This work has parlayed into thoughtful utilization of biomarkers to identify patients early on that are at the highest risk of developing severe AKI (58). Recently, a similar scoring system has been developed in a multicenter cohort of critically ill neonates in India termed the “STARZ” study (59). Utilization of this scoring system allowed for the successful prediction of AKI in the first 7 postnatal days. Further research is needed to develop and validate such scoring tools in other neonatal populations.

#### Nephrotoxic Medication

The contribution of NTX receipt to the incidence of AKI has been increasingly recognized among hospitalized children. This led to the development of the NINJA study designed to mitigate NTX-associated AKI in older children. This was initially studied as a single center experience and validated in a multicenter study in hospitalized children (40, 41). This strategy utilizes the electronic medical record to identify patients at high risk of nephrotoxic AKI (based on receipt of intravenous aminoglycosides ≥3 days or ≥3 NTX given concurrently) and subsequently trigger kidney function monitoring with daily SCr. Stoops et al. has extended this work to critically ill neonates in the “Baby NINJA” study (44). This study identified neonates at high risk of nephrotoxic AKI (defined as ≥3 NTX within 24 h or ≥4 calendar days of an intravenous aminoglycoside) and triggered daily SCr measurement until 2 days after end of exposure or end of AKI. This study showed implementation was feasible and associated with improved AKI metrics including reduced NTX exposure, reduced nephrotoxic AKI, and reduced AKI intensity.

#### Methylxanthine Therapy: Theophylline and Caffeine

Methylxanthines are adenosine-receptor antagonists. In high risk populations methylxanthines have been shown to prevent the development of AKI by preventing adenosine driven pre-glomerular vasoconstriction and post-glomerular vasodilation (60).

Theophylline has been extensively studied in neonates with HIE. There are now nine randomized controlled trials in term neonates with HIE comparing a single dose of theophylline (5–8 mg/kg) vs. placebo (60–67). These studies have consistently shown that theophylline is safe and reduces AKI rates, protects the renal tubule, and improves fluid balance, GFR and urine output. The current KDIGO clinical practice guidelines “suggest that a single dose of theophylline may be given in neonates with severe perinatal asphyxia who are at high risk for AKI” (3). To date there have not been any studies evaluating the impact of theophylline in neonates undergoing therapeutic hypothermia.

In premature neonates, caffeine is often utilized to prevent or treat apnea of prematurity. Carmody et al. first evaluated the impact of caffeine administration during the first postnatal week on the incidence of AKI in a single center cohort of 140 VLBW neonates (68). This study showed that caffeine exposure in the first postnatal week was associated with decreased odds of AKI (aOR 0.22, 95% CI 0.07–0.75; *p* = 0.02). These findings were subsequently confirmed in a secondary analysis of the AWAKEN study (69). More recently, caffeine has been shown to be associated with lower incidence of AKI in neonates with NEC (70).

#### Treatment of Sequelae: Kidney Support Therapy

The indications for KST in neonates are similar to those in older children and include uremia, electrolyte abnormalities, metabolic syndromes, inability to provide adequate nutrition, and the pathologic state of FO. The two modalities of KST commonly utilized in neonates are peritoneal dialysis (PD) and continuous kidney support therapy (CKST). The choice between these two modalities is often driven by clinical scenario, institutional expertise, and available resources. PD is generally less resource intensive and may be performed with the placement of a chronic catheter or a temporary catheter. A detailed discussion of the utilization of PD in AKI has recently been published by the International Society for Peritoneal Dialysis (71).

In recent years there have been extraordinary advances in the ability to provide CKST to critically ill neonates. Prior to this time, CKST was provided utilizing machines and filters that were designed for adults, and significant hemodynamic instability was common and often prohibitive. More recently, smaller filters such as the Prismaflex HF-20 (Baxter Healthcare Corporation, Deerfield, IL; extracorporeal volume 60 mL) have been developed which can be utilized to provide CKST in neonates. One of the most exciting advancements has been the development of CKST devices designed specifically for utilization in neonates. In 2014, Ronco et al. first reported the use of the Cardio-Renal Pediatric Dialysis Emergency Machine (*CARPEDIEM*^®^; Bellco Medtronic, Mirandola, Italy) device (72). Since, then, similar devices include the Nidus^®^ (Allmed, London, England) and the Aquadex^®^ (CHF Solutions, Eden Prarie, MN) have been developed with circuit volumes less than half of that of other available CKST circuits (73, 74). These devices are specifically designed for neonatal patients to decrease hemodynamic instability and improve CKST delivery and tolerance.

## Long-Term Follow Up

In the US, a 21% increase in preterm birth rates was noted from 1980 to 2000 (75–77). Survival rates have similarly improved with those born as early as 25 weeks’ GA now having a >80% chance of survival (78). With these improved survival rates, risks for long-term complications of prematurity, including develop of CKD after AKI, have increased and warrant follow-up. CKD is increasingly prevalent, particularly in infants born <2.5 kg, presenting as decreased renal volume, hypertension (HTN), and/or microalbuminuria (78).

Data from both animal and human models suggest AKI likely leads to permanent kidney damage (78–83). In a review of eight longitudinal studies from 1978 to 2014 examining long-term kidney outcomes following neonatal AKI, rates of CKD in survivors of neonatal AKI were as high at 66% (78). In a follow-up study of AKI in VLBW infants at median age of 5 years, children with a history of AKI had a higher risk of kidney dysfunction than those who never had AKI (65 vs. 14%, RR 4.5 (1.2–17.1); *p* = 0.01) (84). Subjects with kidney dysfunction were more likely to have had a higher stage of AKI, msore episodes of AKI, higher peak SCr, and more days with SCr >1 mg/dl. In a long term follow-up study of extremely premature infants with significant AKI (SCr > 2.0 mg/dl), Abitbol et al. found that at median follow up of 6.6 years, 85% of patients had either a reduced GFR or an elevated urine protein:creatinine ratio (PCR); a SCr > 0.6 mg/dl and urine PCR > 0.6 mg/mg at 1 year of age were most predictive of CKD progression (85). They also found an association with body mass index (BMI) > 85th percentile at 3 years of age and increased risk of kidney dysfunction.

Although current evidence of significant risk of CKD following neonatal AKI is largely based on small observational studies, it is expert opinion that all neonates with an identified AKI episode should have longitudinal follow-up (78). KDIGO guidelines recommend patients with a history of AKI have a CKD evaluation 3 months after their AKI event (3). Aside from this and recommendations from the American Academy of Pediatrics to begin blood pressure monitoring earlier in infants born LBW (prior to the typical 3 years of age in infants born at term), there are currently no evidence-based guidelines governing who should follow neonates after AKI or how frequently they should be screened for HTN and CKD (86). Chaturvedi et al. recommends screening of all patients with history of AKI for HTN and albuminuria at least annually, with more invasive testing (e.g., SCr) recommended for patients at higher risk (e.g., more severe AKI, KDIGO stages 2 and 3) (78). Counseling should also be performed regarding healthy weight and lifestyle choices given the association between elevated BMI and kidney dysfunction. These recommendations underscore the importance of proper AKI diagnosis in the neonatal period to identify patients in need of follow-up prior to discharge from the NICU.

## Conclusive Results

Neonatal AKI is prevalent, particularly in high risk populations, including those born prematurely or with LBW, those with congenital heart disease, HIE, NEC, and in neonates who receive NTX or require ECLS. With ever-increasing study and the utilization of the expert-endorsed, modified, neonatal KDIGO criteria, the consequences of neonatal AKI are becoming clear. Identification of those infants at highest risk for AKI using protocolized surveillance and novel biomarkers is an area of active study. Avoidance of NTX and treatment of specific, at-risk populations with methylxanthines may improve AKI rates or mitigate AKI that has already occurred. With a lack of specific treatments currently available, prevention and prompt diagnosis are key. Emerging evidence suggests innovative KST technologies may improve survival and that long-term follow-up is necessary given the risk of HTN and potentially CKD after neonatal AKI.

## Author Contributions

DS, CC, ATP, and HS each developed the abstract, developed the outline, and wrote specific sections of the manuscript. All authors edited the final manuscript.

## Conflict of Interest

The authors declare that the research was conducted in the absence of any commercial or financial relationships that could be construed as a potential conflict of interest.

## Publisher’s Note

All claims expressed in this article are solely those of the authors and do not necessarily represent those of their affiliated organizations, or those of the publisher, the editors and the reviewers. Any product that may be evaluated in this article, or claim that may be made by its manufacturer, is not guaranteed or endorsed by the publisher.

## Abbreviations

AKI: acute kidney injury
NICU: neonatal intensive care unit
CKD: chronic kidney disease
KST: kidney support therapy
KDIGO: Kidney Disease: Improving Global Outcomes
AWAKEN: Assessment of Worldwide Acute Kidney Injury Epidemiology in Neonates
SCr: serum creatinine
GA: gestational age
HIE: hypoxic ischemic encephalopathy
ACE: angiotensin converting enzyme
NSAIDs: non-steroidal anti-inflammatory drugs
kg: kilograms
BW: birth weight
LBW: low birth weight
GFR: glomerular filtration rate
aOR: adjusted odds ratio
CI: confidence interval
VLBW: very low birth weight
FO: fluid overload
NTX: nephrotoxic medication
MRI: magnetic resonance imaging
NEC: necrotizing enterocolitis
IQR: interquartile range
NINJA: Nephrotoxic Injury Negated by Just-in-time Action
ECLS: extracoporeal life support
KIDMO: Kidney Intervention during Extracorporeal Membrane Oxygenation
IVH: intraventricular hemorrhage
BPD: bronchopulmonary dysplasia
RR: relative risk
NGAL: neutrophil gelatinase associated lipocalin
PD: peritoneal dialysis
CKST: continuous kidney support therapy
PCR: protein-to-creatinine ratio
BMI: body mass index
HTN: hypertension
tx: treatment.
